# Longitudinal leisure-time physical activity profiles throughout adulthood and related characteristics: a 36-year follow-up study of the older Finnish Twin Cohort

**DOI:** 10.1186/s12966-024-01600-y

**Published:** 2024-04-26

**Authors:** Bram J. Berntzen, Asko Tolvanen, Urho M. Kujala, Karri Silventoinen, Eero Vuoksimaa, Jaakko Kaprio, Sari Aaltonen

**Affiliations:** 1 Institute for Molecular Medicine Finland FIMM, University of Helsinki, P.O. Box 20, FI-00014 Helsinki, Finland; 2 Methodology Centre for Human Sciences, University of Jyväskylä, P.O. Box 35, FI-40014 Jyväskylä, Finland; 3 Faculty of Sport and Health Sciences, University of Jyväskylä, P.O. Box 35, FI-40014 Jyväskylä, Finland; 4 Helsinki Institute for Demography and Population Health, University of Helsinki, P.O. Box 42, FI-00014 Helsinki, Finland

**Keywords:** Exercise, Latent classes, Longitudinal, Sports, Working-age individuals

## Abstract

**Background:**

Personalized interventions aiming to increase physical activity in individuals are effective. However, from a public health perspective, it would be important to stimulate physical activity in larger groups of people who share the vulnerability to be physically inactive throughout adulthood. To find these high-risk groups, we identified 36-year leisure-time physical activity profiles from young adulthood to late midlife in females and males. Moreover, we uncovered which anthropometric-, demographic-, lifestyle-, and health-related characteristics were associated with these physical activity profiles.

**Methods:**

We included 2,778 females and 1,938 males from the population-based older Finnish Twin Cohort Study, who responded to health and behavior surveys at the mean ages of 24, 30, 40 and 60. Latent profile analysis was used to identify longitudinal leisure-time physical activity profiles.

**Results:**

We found five longitudinal leisure-time physical activity profiles for both females and males. Females’ profiles were: 1) *Low increasing moderate* (29%), 2) *Moderate stable* (23%), 3) *Very low increasing low* (20%), 4) *Low stable* (20%) and 5) *High increasing high* (9%). Males’ profiles were: 1) *Low increasing moderate* (29%), 2) *Low stable very low* (26%), 3) *Moderate decreasing low* (21%), 4) *High fluctuating high* (17%) and 5) *Very low stable* (8%). In both females and males, lower leisure-time physical activity profiles were associated with lower education, higher body mass index, smoking, poorer perceived health, higher sedentary time, high blood pressure, and a higher risk for type 2 diabetes. Furthermore, lower leisure-time physical activity was linked to a higher risk of depression in females.

**Conclusions:**

We found several longitudinal leisure-time physical activity profiles with unique changes in both sexes. Fewer profiles in females than in males remained or became low physically active during the 36-year follow-up. We observed that lower education, higher body mass index, and more smoking already in young adulthood were associated with low leisure-time physical activity profiles. However, the fact that several longitudinal profiles demonstrated a change in their physical activity behavior over time implies the potential for public health interventions to improve leisure-time physical activity levels.

**Supplementary Information:**

The online version contains supplementary material available at 10.1186/s12966-024-01600-y.

## Introduction

Long-term physical inactivity in adulthood is a major global health challenge [1], with a substantial economic burden [2]. The physical inactivity related health challenge can partly reflect the findings of previous research that have established heterogeneity and low stability in physical activity (PA) behavior but higher stability in physical inactivity behavior during the life course [3]. Personalized interventions that aim to increase PA could be an effective way to improve health and well-being in adulthood [4]. However, at the population level, it would be more valuable to increase leisure-time PA (LTPA) in larger groups of people who share similar longitudinal LTPA profiles or trajectories during the life course and then tailor interventions by the unique characteristics of these groups.

Although PA research increasingly addresses longitudinal PA profiles and trajectories, so far, only a minority of studies have tracked PA over multiple decades. A systematic review of group-based PA trajectories was published in 2019 and included 27 studies [5]. Of these studies, four had a follow-up duration of over three decades which addressed two different cohorts: 1) the Cardiovascular Risk in Young Finns Study (YFS) [6, 7] and 2) the Northern Finland Birth Cohort [8, 9]. These studies with baseline measurements in 1980 uncovered three to five PA trajectories and showed a high proportion of physical inactivity at all ages, which further increased with aging. Published after that review, another 31-year follow-up study in the YFS of children and adolescents aged 9 to 18 at baseline found that about two-thirds of the participants belonged to trajectories with low LTPA levels at the end of follow-up [10]. Recently, Norwegian researchers focused on vigorous LTPA in their 27-year follow-up study [11]. They found four trajectories of vigorous LTPA between ages 13 and 40 years. In order to cover overall PA over the entire lifespan, one study investigated older adults who recalled their PA in young, middle, and older adulthood [12]. This retrospective study found 6 life-course PA trajectories; 73% of adults were identified in trajectory classes that indicated persistent low PA and 13% in trajectory classes showed a large decrease in PA over time.

Even fewer studies have tried to uncover whether the PA profiles or trajectories over three decades differ between females and males. The existing studies have suggested that more females belong to low PA trajectory classes than males [6, 10]. These results also reflect those of a trajectory study of a somewhat shorter follow-up (i.e., 24 years) [13]. When females and males have been studied separately, consistently high and increasing LTPA trajectories have been linked to healthier diets and less smoking in both sexes [10], as well as to the absence of sleep difficulties [10] and less sedentary behavior measured as television time in females [14]. In studies combining females and males, low or decreasing PA trajectories over three decades have been shown to be associated with low education [6], smoking [6] and depressive symptoms [7]. Studies combining females and males in PA profiles or trajectories with shorter follow-ups – between 3 and 27 years – have shown that low and decreasing PA profiles and trajectories are associated with lower socioeconomic status [5], lower income [11], poorer diet [15, 16], lower alcohol consumption [15–17], smoking [15], poorer subjective health [15, 17], cognitive decline [16, 18], the onset of depression [7], higher body mass index (BMI) [16, 17, 19], incidence of type 2 diabetes (T2D) [20], and an increased risk of cardiovascular disease [21]. Previous evidence also suggests that the health benefits of LTPA may depend on occupational PA [22].

Considering all of this evidence, it seems that only the 31-year YFS study focusing on LTPA from childhood to early midlife has tried to uncover different PA trajectories of more than three decades and simultaneously examine how multiple anthropometric, demographic, and lifestyle factors are associated with these trajectories in females and males separately [10]. Thus, a longitudinal profile or trajectory study covering LTPA over the course of working life is still lacking. However, this would be a key life phase to intervene upon, not only to reduce health complications that come with long-term physical inactivity and aging, but also to improve productivity at work [23]. Therefore, our aim is to identify 36-year LTPA profiles for females and males from young adulthood to late midlife. Furthermore, we aim to uncover which anthropometric-, demographic-, lifestyle-, and health-related characteristics are associated with these longitudinal LTPA profiles.

## Methods

### Participants

The participants of this study were from the older Finnish Twin Cohort study, which is a longitudinal population-based study of twins from same-sex pairs born in Finland before 1958 (N=13,888) [24, 25]. Twins responded to health survey questionnaires in 1975, 1981, 1990 and 2011 (response rates from 89% to 72%). The research was conducted according to the principles of the Declaration of Helsinki, and the data collection was approved by the ethics committee of the Hjelt Institute, University of Helsinki and the ethics committee of the Helsinki and Uusimaa Hospital District, Finland. All participants gave informed consent.

To create longitudinal LTPA profiles from young adulthood to late midlife, we used data from all four follow-ups. We included those twins who were between ages 18–31 (mean age 24.1 years; *N*=11,921 individuals) at baseline in 1975. In 1981 and 1990, the participants were at the mean ages of 30.3 years (age range 23–39; *N*=10,689 individuals) and 39.5 years (age range 32–47; *N*=7,473 individuals), respectively. At the last follow-up in 2011, the participating twins had reached the mean age of 60.2 years (age range 53–67; *N*=7,381 individuals). Hereafter the mean ages are referred to as 24, 30, 40 and 60. In total, we had LTPA data at all time points available from 4,716 twin individuals (2,778 females and 1,938 males), including 1,328 complete twin pairs.

### Assessment of leisure-time physical activity

Participants reported their LTPA, which was quantified as metabolic equivalent of task (MET) hours expended per day. These values were based on a series of structured and validated items on the frequency, mean duration and mean intensity of LTPA sessions, as well as an item on commuting activity [26, 27]. To calculate MET hours per day, we used the following formula: LTPA frequency (average per day) × LTPA duration (average hours) × LTPA intensity (activity MET score) [28]. The following MET values were used for the intensity of LTPA to obtain a multiple of the resting metabolic rate for each activity: 4 corresponded to walking, 6 corresponded to vigorous walking to jogging, 10 corresponded to jogging, and 13 corresponded to running. The MET value of 4 (walking) was also used for the intensity of commuting-related PA. We further assumed that commuting-related PA was done on 5 days per week. All types of LTPA and commuting-related physical activities were considered when MET hours per day were calculated.

The LTPA items were included in all survey questionnaires in the same form, except in 1990 when only one item was used. This item measures combined information on the frequency, duration and intensity of LTPA, including commuting activity. We converted this item to MET-hours/day as well.

### Assessment of demographic, anthropometric and lifestyle characteristics

To investigate the associations between longitudinal LTPA profile membership and various characteristics, we selected a group of characteristics, that have been shown to be related to different longitudinal PA profiles and trajectories in previous studies (details given in the introduction) [5–7, 10, 11, 13, 15–22, 29, 30]. The selection of these characteristics was also based on prior large systematic reviews, meta-analyses and guidelines, indicating the disease, health, and lifestyle correlates of PA and physical inactivity behaviors [31–34]. Scientific evidence on the importance of some lifestyle-related characteristics (e.g., sitting) has become available only in recent decades and, therefore, such characteristics were only available from the last follow-up survey questionnaire in 2011.

At baseline, the participants reported their financial situation by responding to a structured survey item with an 8-point Likert scale on their monthly income. Higher scores indicate higher monthly income. The measure of education was the self-reported highest educational degree achieved at ages 24 and 30, based on the 1975 and 1981 surveys. The eight categories of level of education ranged from less than compulsory education (1) to tertiary education (i.e., university or polytechnic college) (8) [35]. The participants reported their height and weight in all surveys, and BMI was calculated as the ratio between weight in kilograms and height in square meters (kg/m^2^). Self-reported BMI has been validated in this cohort [36, 37]. At age 60, the participants were also asked to measure their waist circumference with a measuring tape sent along with the survey questionnaire.

Regarding lifestyle characteristics, alcohol consumption, smoking and sleeping were reported by the participants at all time points. The number of monthly alcoholic beverages participants reported to drink was converted into grams of 100% alcohol per month [38, 39]. Smoking status (never/former/current) was defined using responses to two dichotomized (yes/no) items on [40]: 1) the history of ever smoking more than 5 - 10 packs of cigarettes and 2) current or previous daily smoking. The structured response options for sleep time were as follows [41]: 1) 7-point Likert scale from <4 hours (1) to >10 hours per night (7) at baseline and 2) 9-point Likert scale from <6 hours (1) to >10 hours per night (9) at all other follow-up time points. Work-related PA data were collected at baseline and the last follow-up time point. The initial 4-category variable of the physical strain of work was used to create a dichotomized variable for the purpose of analysis: 1) sedentary work that may involve walking and 2) manual work that may involve lifting and carrying heavy objects. At the last follow-up time point, participants also reported how many hours they were sitting (i.e., sedentary behavior) per day 1) in an office, 2) while watching TV or videos at home, 3) at a computer at home, 4) in a vehicle, and 5) elsewhere. The 4 response options given for sitting ranged from <1 hour (1) to >4 hours per day (4), and a sum score of the distinct sitting categories were used to create a final sedentary behavior variable (score ranged 1–20) with higher scores indicating higher overall sitting times [42].

### Assessment of health characteristics

At the last follow-up time point, the participants rated how they perceived their health in general. The 5-point Likert scale ratings ranged from “very good” (1) to “very poor” (5). For data analysis, we dichotomized the subjective health variable so that the response options “very good” and “good” were defined as “good” (1), while the rest of the response options were defined as “poor” (2). In addition to this general health item, the participants also reported whether they had ever been diagnosed by a physician (yes/no) with: high blood pressure (all follow-ups), T2D (all follow-ups), coronary artery disease, including angina pectoris (all follow-ups), or depression (last follow-up).

### Statistical analysis

In order to identify different longitudinal LTPA profiles from young adulthood to late midlife, we used the latent profile analysis, which can be considered as a subset of finite mixture models [43]. In the latent profile analysis, researchers do not impose growth trends on the data. Rather, the profiles are a direct reflection of the data making them a more accurate description of the profiles. We classified participants into distinct profiles based on the means and variances of their LTPA behavior (i.e., there were different means and variances between the identified profiles). The correlation between LTPA variables was set to zero because local independence is the basic assumption underlying the latent profile analysis. Eventually, this analysis of 36-year LTPA behavior across four time points led to longitudinal profiles in which individuals within a profile were more similar than individuals between profiles.

Using this approach, we first estimated and compared models with differing numbers of latent profiles to determine which of the models fit the data best, using the Bayesian information criteria (BIC) and Lo-Mendel-Rubin adjusted test (LMR). The lower the BIC value is, the better the model fits the data, while a p-value < 0.05 in the LMR test is used as an indicator to reject the model with fewer profiles (i.e., k number of latent profiles fits the data better than k-1 number of latent profiles). For the best fitting model, we calculated entropy and average latent profile probabilities, indicating the distinctiveness between profiles. We conducted the analyses separately for females and males. If the results suggested an equal number of latent profiles for females and males, we continued our analyses by comparing the profiles between the sexes in successive steps: (1) the equality of mean values, (2) the equality of variances and (3) the equality of latent profile sizes [44].

We estimated all the models using the maximum likelihood method with the Mplus 8.7 statistical package [45]. The maximum likelihood estimation with robust standard errors (robust to non-normality) was utilized. After the final number of LTPA profiles were identified, we continued analyses by examining associations between latent profile membership and various demographic, anthropometric, lifestyle and health characteristics using the one-step Bolck-Croon-Hagenaars method for continuous variables and the two-step model-based approach test for categorical variables [46, 47]. Regarding these association results, the p-value of < 0.001 corresponds to a multiple-test corrected Bonferroni p-value < 0.05 (i.e., 0.05/45 tests=0.001). Moreover, because we had complete twin pairs in our data, the non-independency of data was possible (the observations and their error terms between the co-twins of a twin pair can be correlated). Therefore, we used the type=complex-option to calculate unbiased standard errors and p-values when examining the associations between latent profile membership and demographic, anthropometric, lifestyle and health characteristics.

## Results

The descriptive statistics of leisure-time physical activity, demographic, anthropometric, lifestyle, and health characteristics are given by sex and age in Supplementary Table 1. We identified 6-profile models to be the best models for both females and males based on the BIC values (Table 1). However, according to the LMR test, for both sexes these 6-profile solutions did not fit better than 5-profile solutions. A closer inspection also further revealed that the 6-profile solution divided one of the 5-profile solutions into two profiles that were equal in their shapes both in females and males. Therefore, to follow the general statistical principle of parsimony, we chose the 5-profile solution as final models for both females and males. Because these results suggested an equal number of profiles for females and males, we further statistically tested the similarity of the 5-profile solutions between females and males. The BIC value for the freely estimated model increased from 89975.01 to 90033.30, when setting latent profile means equal. This indicated that latent profile solutions needed to be analyzed separately for females and males.

**Table 1:** The comparison of models with differing numbers of latent profiles to determine which of the models fits the data best

Number of latent profiles	BIC	LMR	Entropy	AvePP	Profile sizes
Females					
1	55979.93	NA	1.0	1.0	2778
2	50078.58	< 0.001	0.838	0.963; 0.939	2149; 629
3	48693.20	< 0.001	0.790	0.873; 0.924; 0.931	777; 1565; 436
4	48250.09	0.002	0.725	0.746; 0.848; 0.946; 0.876	542; 752; 367; 1117
5	47914.81	0.002	0.690	0.798; 0.802; 0.931; 0.830; 0.760	792; 553; 256; 628; 549
6	47743.52	0.078	0.695	0.754; 0.794; 0.841; 0.785; 0.912; 0.815	524; 499; 197; 804; 155; 599
Males					
1	43138.70	NA	1.0	1.0	1938
2	37605.22	< 0.001	0.857	0.958; 0.961	670; 1268
3	36391.14	< 0.001	0.840	0.906; 0.953; 0.934	445; 510; 983
4	36002.79	0.095	0.745	0.882; 0.797; 0.804; 0.956	435; 544; 530; 429
5	35601.98	0.007	0.773	0.846; 0.926; 0.838; 0.929; 0.835	502; 147; 563; 321; 405
6	35284.26	0.167	0.765	0.917; 0.788; 0.828; 0.932; 0.823; 0.827	113; 315; 458; 247; 474; 331

*BIC* Bayesian information criteria, *LMR* Adjusted Lo-Mendell-Rubin likelihood ratio test, *AvePP* Average latent profile probabilities for the most likely latent

profile membership

### Longitudinal LTPA profiles

**Fig. 1:**
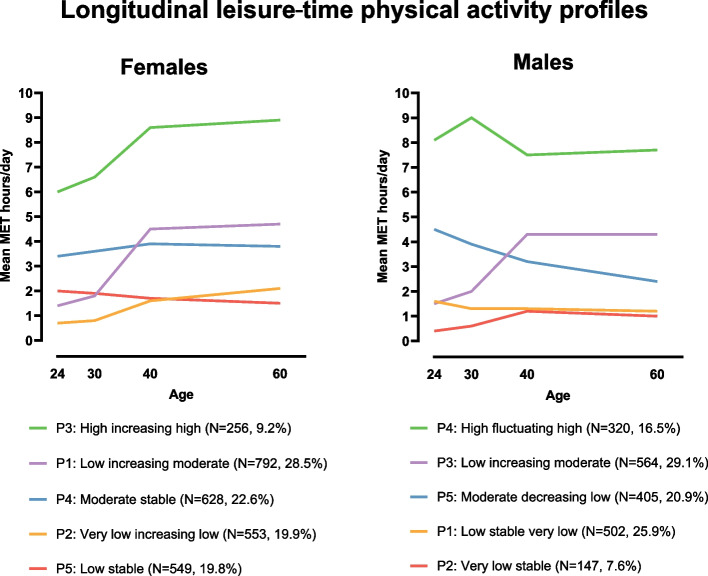
Longitudinal leisure-time physical activity profiles from young adulthood to late midlife in females and males. MET=metabolic equivalent of task

We labeled the longitudinal LTPA profiles based on their unique elements 1) at baseline, 2) during the transition toward the final time point, and 3) at the end of follow-up (Fig. 1 and Supplementary Table 2). The largest proportions of both females (*n*=792, 29%; Profile 1) and males (*n*=564, 29%; Profile 3) belonged to *Low increasing moderate* profiles. As our labeling indicates, these profiles in both sexes were characterized by low LTPA levels at baseline, then increasing their LTPA levels first slightly and then more steeply to a mean of 4.5 (females) and 4.3 (males) MET hours/day. The second largest proportion of females (*n*=628, 23%) was assigned to Profile 4, *Moderate stable*, characterized by a stable level of LTPA (between 3.4 and 3.9 MET hours/day) throughout the entire follow-up. In males, the corresponding moderate-level LTPA profile was Profile 5 (*n*=405, 21%) that we labeled as *Moderate decreasing low.* This profile was characterized by a steady LTPA decrease over time (from mean 4.5 MET hours/day to 2.4 MET hours/day). The third largest proportions of females belonged to Profile 2 (*n*=553, 20%), *Very low increasing low*. These women had a mean MET hours/day of 0.7 at baseline, slightly increasing from age 30 to reach the mean level of 2.1 MET hours/day at the end of the follow-up. In males, the corresponding profile, but with an opposing trend, was Profile 1 (*n*=502, 26%), *Low stable very low*, which remained fairly stable with a minimal decrease, going from 1.6 MET hours/day to 1.2 MET hours/day. The fourth largest female LTPA group was Profile 5 (*n*=549, 20%), *Low stable.* This profile was characterized by a stable low level of LTPA (between mean 2.0 and 1.5 MET hours/day) throughout the follow-up, having the lowest LTPA level at the end of follow-up. In males, the profile with the lowest LTPA at all timepoints was Profile 2 (*n*=147, 8%), *Very low stable*. This LTPA profile portrayed a very inactive group of young male adults (mean 0.4 MET hours/day), who slightly increased their LTPA level, but kept it fairly constant until the end of follow-up (at the highest point the mean level was 1.2 MET hours/day). The smallest proportion of females (n=256, 9%) belonged to the profile of the highest level of LTPA, Profile 3, *High increasing high*. Females in this profile were those with the highest LTPA level in young adulthood (mean 6.0 MET hours/day), and they even increased their LTPA level over time (mean 8.9 MET hours/day). The corresponding high-level LTPA profile in males was Profile 4 (*n*=320, 17%), *High fluctuating high.* These males consistently kept their mean MET hours/day over 7.4, despite some fluctuation over time.

### Longitudinal LTPA profile associations with demographic and anthropometric characteristics

**Fig. 2:**
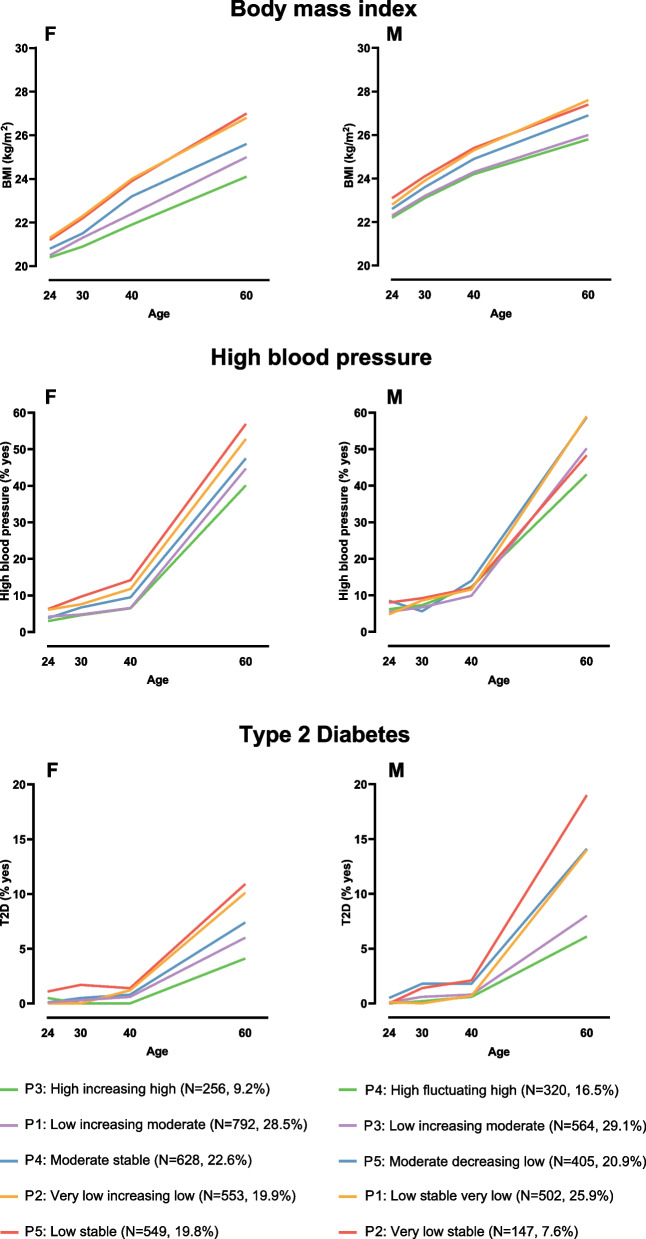
Mean body mass index, percentages of individuals with high blood pressure and percentages of individuals with type 2 diabetes from young adulthood to late midlife by longitudinal leisure-time physical activity profiles. Left panels (F) represent females and right panels (M) males. BMI=body mass index; kg=kilogram; m=meter; T2D=type 2 diabetes

**Fig. 3:**
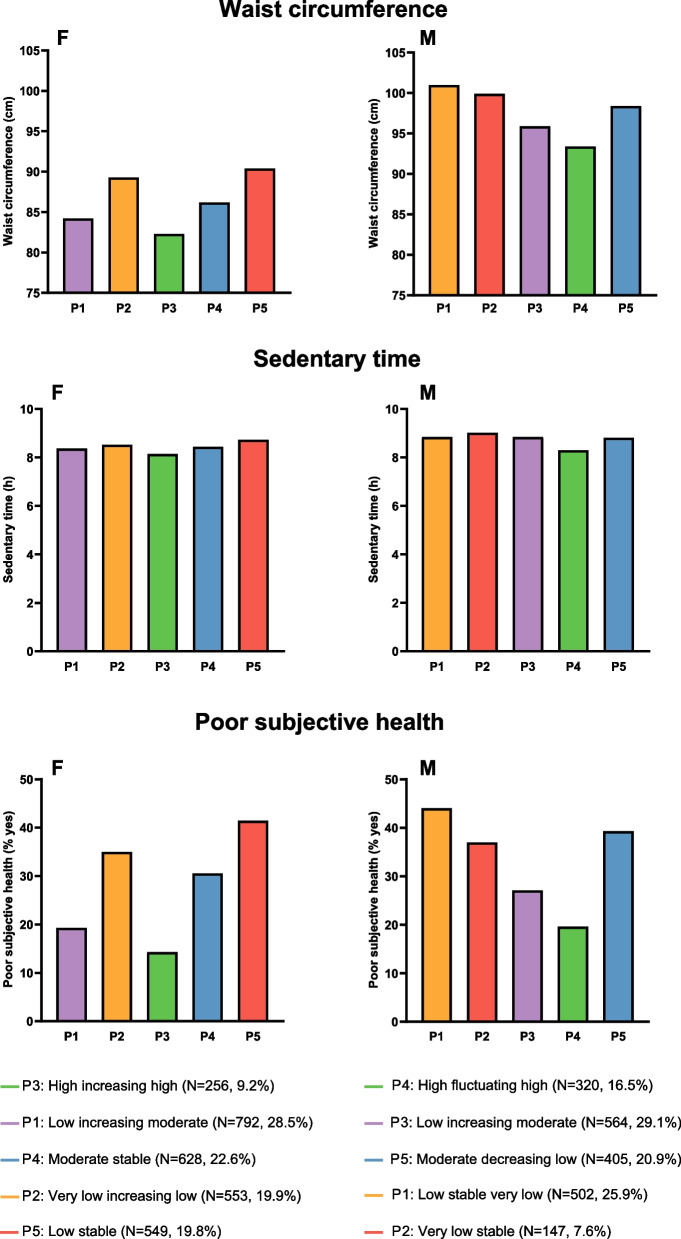
Mean waist circumference, mean sedentary time and percentages of individuals with poor subjective health in late midlife (i.e., the last follow-up at age 60) by longitudinal leisure-time physical activity profiles. Left panels (F) represent females and right panels (M) males. cm=centimeter; h=hours

**Fig. 4:**
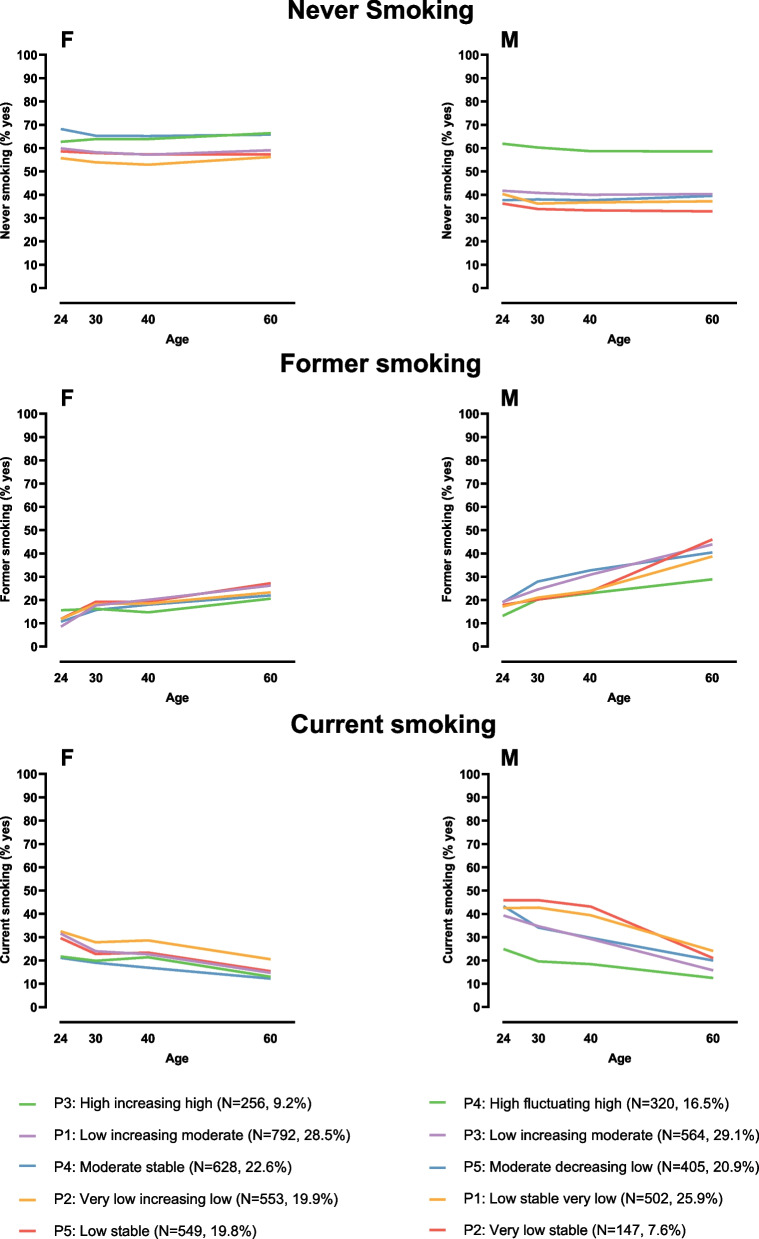
Percentages of never, former and current smokers from young adulthood to late midlife by longitudinal leisure-time physical activity profiles. Left panels (F) represent females and right panels (M) males

The results of the comparisons of demographic, anthropometric and lifestyle characteristics between longitudinal LTPA profiles in females and males are presented in Figs. 2, 3, 4 and Supplementary Tables 3 and 5. Although we detected no significant age or income differences between the five longitudinal LTPA profiles in either females or males, educational differences between profile memberships were found. Females in the *Very low increasing low* profile were significantly more likely to have lower education relative to those in the *Low increasing moderate* (*p*-value=0.007), *Moderate stable* (*p*=0.001), and *Low stable* profiles (*p*=0.019) at age 24, as well as to all other profiles at mean age 30. Males in the *Very low stable* profile reported significantly lower educational attainment than those in other profiles (p-values <0.001). The members of the *Low stable very low* and *Low increasing moderate* profiles also reported somewhat lower educational attainment than those who belonged to the *High fluctuating high* profile (*p*-values <0.011).

For weight and BMI, significant differences were detected by longitudinal LTPA profiles throughout the follow-up in females (*p*<0.001): those females in the *High increasing high* profile had the lowest body weight and BMI at each time point, while those in the *Very low increasing low* and *Low stable* profiles consistently had the highest body weight and had the highest mean BMI. The same pattern occurred in males, with a significantly higher body weight and BMI in the two lowest LTPA profiles, *Low stable very low* and *Very low stable*, compared to the highest LTPA profiles, *High fluctuating high* and *Low increasing moderate* (*p*-values <0.027). Moreover, waist circumference measured at the last follow-up time point was smaller among those females who belonged to the *High increasing high* and *Low increasing moderate* profiles (*p*-values <0.001). Males in the *High fluctuating high* profile also had a significantly smaller mean waist circumference at the last follow-up time point compared to other profiles (p-values <0.003). These anthropometric differences between longitudinal LTPA profiles remained significant in females after Bonferroni correction (Supplementary Table 3).

### Longitudinal LTPA profile associations with lifestyle characteristics

Regarding lifestyle characteristics, females and males in different longitudinal LTPA profiles had similar alcohol consumption habits within their own sex groups, with the exception of age 40 when males in the *Low increasing moderate* profile used less alcohol than male individuals in the *Low stable very low* (*p*=0.010) and *Very low stable* profiles (*p*=0.035). Females in the *Moderate stable* profile more likely had never smoked compared to those females in profiles who started off with low LTPA at age 24 (*p*-values <0.047), but no differences appeared at age 30 (Fig. 4). In females at age 40 and 60, the *Very low increasing low* profile had the most current female smokers, while the *Moderate stable profile* had the fewest (*p*<0.001 at age 40 and *p*=0.005 at age 60). In terms of male smoking, there were significantly more males in the *High fluctuating high* profile who were never smokers and fewer current smokers compared to other profiles at all time points (*p*-values <0.001 and <0.010, respectively). By age 60, the *High fluctuating high* profile had also the lowest proportion of former male smokers (*p*-values <0.024). Differences in sleep did not emerge between longitudinal LTPA profiles in males, whereas females in the *Very low increasing low* profile slept significantly more per night than females in all other profiles (*p*=0.022) at age 30.

We did not find any differences in work-related PA levels between the longitudinal LTPA profiles but sedentary behavior (i.e., sitting) differences at age 60 existed. Females in the *Low stable* profile sat significantly longer per day than those in *Low increasing moderate* (*p*=0.018) and *High increasing high* profiles (*p*=0.001), and females in the *Very low increasing low profile* sat longer than those in the *High increasing high* profile (*p*=0.034). Similarly, males in the *High fluctuating high* profile also reported significantly less time spent in sedentary behavior per day than those in other profiles (*p*=0.003).

### Longitudinal LTPA profile associations with health characteristics

The associations between females’ and males’ longitudinal LTPA profiles and different health characteristics are shown in Figs. 2, 3, 4 and Supplementary Tables 4 and 6. The subjective health status was assessed after the 36-year follow-up and females differed significantly between the longitudinal LTPA profiles (*p*<0.001): those in the *Low increasing moderate* and *High increasing high* profiles reported overall better health statuses than other LTPA profiles. Males in the *High fluctuating high* profile reported the highest subjective health status compared to other longitudinal LTPA profiles (*p*-values <0.042).

At ages 24 and 30, no differences in high blood pressure appeared in females. However, as time passed, the *Low increasing moderate* and *High increasing high* profiles were significantly less likely to have high blood pressure than their counterparts in the *Very low increasing low* and *Low stable* profiles at ages 40 and 60 (*p*-values <0.026), with the exception of the difference between the *High increasing high* and *Very low increasing low* profiles at age 40. Even then, the difference almost reached statistical significance (*p*=0.053). Males aged 60 in the *Low stable very low* profile more likely had high blood pressure than all other profiles, except the *Moderate decreasing low* profile (*p*-values <0.044). Males in the *Moderate decreasing low* profile also more often had high blood pressure than the *High fluctuating high* profile (*p*=0.001). No differences in coronary artery disease emerged between longitudinal female LTPA profiles, whereas fewer coronary artery disease cases were reported among males in the *High fluctuating high* profile compared to most other male LTPA profiles at age 24 (*p*-values <0.029).

Females in the *Very low increasing low* profile had the lowest prevalence of T2D (*p*<0.001) at baseline, but from age 40 onward, fewer T2D cases were reported by the *Low increasing moderate* and *High increasing high* profiles compared to the *Very low increasing low* (*p*-values <0.038) and *Low stable* profiles (*p*-values <0.022). Comparably, in males, fewer T2D cases were reported among males in the *Very low stable* profile at age 24, but at age 60, males in the *Low increasing moderate* and *High fluctuating high* profiles had a significantly lower T2D prevalence than those in other profiles (*p*-values <0.031). At the end of the follow-up, significantly fewer cases of depression were reported among females in the *Low increasing moderate* and *High increasing high* profiles than those in the *Moderate stable* and *Low stable* profiles (*p*-values <0.042). No differences in depression appeared between longitudinal LTPA profiles in males.

## Discussion

To our knowledge, this is the first study assessing longitudinal LTPA profiles with an agnostic approach over the course of working life. We identified 36-year LTPA profiles from young adulthood to late midlife, and uncovered which anthropometric-, demographic-, lifestyle-, and health-related characteristics were associated with these longitudinal LTPA profiles. We found five longitudinal LTPA profiles in both females and males with a unique magnitude, rate, and timing of LTPA changes. Overall, we discovered that about half of the participants belonged to the longitudinal profiles that were stable (females 42% and males 50%), and the rest belonged to the longitudinal profiles that changed over time. These changing profiles were increasing (58%) in females, and fairly evenly distributed between increasing (29%) and decreasing (21%) in males. Two of the longitudinal profiles in females (40% of females) and three in males (54% of males) remained or became inactive over time. Changes in longitudinal LTPA profiles occurred most at ages 30 and 40. Our results also revealed that the longitudinal LTPA profiles that were at lower levels over the follow-up period were associated with lower education, higher weight, higher BMI, smoking, poorer perceived health, higher sedentary time, high blood pressure, and the higher risk of T2D in both females and males. Furthermore, the longitudinal profiles with lower LTPA levels were associated with more depression in females.

Although longitudinal LTPA profiles were distinct in females and males, there were also similarities. Thus, low LTPA levels were the most common in young adulthood, and the longitudinal LTPA profiles with the largest proportion of individuals started with low LTPA levels in young adulthood, then increased to moderate LTPA levels by age 40, and maintained that level until the end of the follow-up. Only one LTPA profile in each sex was identified to be the high LTPA level profile – the smallest proportion of participants (9%) in females and the second smallest proportion in males (17%). In line with previous long-term (>30-year follow-up) LTPA trajectory studies [6, 10], we found that males were more likely to have high LTPA levels than females until midlife. This may reflect a true difference between sexes, but one explanation for these results could also be that LTPA questionnaires may focus more on male-oriented physical activities. Reproduction and family life may explain some of the longitudinal LTPA profile differences between females (having more children often means less time for LTPA), but also between females and males because this affects females more strongly than males (e.g., child-bearing and caring for children) [48]. On the other hand, males may have had more career demands and responsibilities to provide for the family in the previous decades, which could have affected their LTPA in our study. Nowadays, most Finnish women are in the workforce, and women may have more difficulties balancing work and family than men even when day care is widely available and used in families [49]. Despite such challenges, an interesting finding was that none of the longitudinal female LTPA profiles notably decreased over the follow-up time. Because nearly 60% of the females had an increasing LTPA trend over time, most females were identified to be part of the longitudinal profiles that ended up with moderate LTPA levels at age 60. For males, longitudinal profiles with low LTPA levels remained the predominant behavior by age 60.

We also wanted to uncover which characteristics could distinguish the longitudinal LTPA profiles. None of the profiles were associated with income, unlike in a previous study [11]. The LTPA profiles starting with very low LTPA in females and males had the lowest education of all LTPA profiles, which was consistent with previous suggestions that higher education may slow the aging-related PA decline [50]. The lowest education levels were found among those females and males who were identified to be part of the very low and low LTPA profiles.

Regarding lifestyle, we found that the participants in the highest and lowest LTPA profiles were the least and most likely to have ever smoked, respectively. In the YFS cohort, it was also shown that people in the highest LTPA trajectories were the least likely to belong to regular smoking trajectories [51]. However, we were not able to replicate the common finding that higher PA trajectories go along with higher alcohol consumption [15–17]. Participants in the highest longitudinal LTPA profiles were also less sedentary at age 60. In addition to the negative health consequences of low LTPA, sedentary behavior has been shown to be independently linked to poor health [52]. This was also the case in our study because the participants in those longitudinal LTPA profiles that sat more reported poorer subjective health. Our present results on sleep also support the previous findings, indicating that differences in sleep duration were absent or minor between LTPA trajectory classes in females and males [10].

Similar to the results of the previous studies [17], we found that females and males in the highest longitudinal LTPA profiles and in the profiles that increased toward moderate LTPA over time had better perceived health status than others. An interesting detail is that those females who belonged to the *Low increasing moderate* profiles reported significantly better subjective health status than females who belonged to the *Moderate stable* profile, even though females in both of these profiles reported the moderate level of LTPA at age 60 when subjective health was assessed. Thus, it seems that the increasing long-term pattern from low LTPA level to moderate LTPA level did matter. In terms of physical activity promotion actions, this would be good news if our result would be a proof of the health effects of LTPA. However, by using only our data and methods, we cannot know about the direction of the association between LTPA and health. Previous longitudinal evidence on models that simultaneously consider both directions of the association has suggested a bidirectional association between these two factors: higher PA positively predicts the better subsequent health, but at the same time high perceived health status seems to predict the high level of future physical activity as well [53, 54].

The highest longitudinal LTPA profiles in both females and males acquired the lowest body weight and BMI consistently over time, as well as the smallest waist circumference at age 60. PA has been shown to be associated with lower adiposity [34], but it appears ineffective for weight loss [55], so PA may support weight gain prevention. These results are in accordance with earlier observations showing that high PA trajectories appeared to go hand in hand with low BMI trajectories in adulthood [19]. However, even in people living with obesity, higher LTPA may be associated with a lower risk of high blood pressure [56]. We found that those females and males who belonged to the highest longitudinal LTPA profiles were less likely to have high blood pressure. In females, this association was found from age 40 onward, whereas in males it was only at age 60. Our study also revealed that those participants who belonged to the high longitudinal LTPA profiles or to the profiles that increased toward moderate LTPA over time reported lower T2D prevalence than the participants in the lower LTPA profiles by age 60. This agrees with a systematic review and meta-analysis of 28 prospective studies showing a benefit of a higher LTPA on T2D incidence [57]. In contrast to an earlier study suggesting a relationship between higher PA trajectories and less major coronary artery disease [21], we found such a relationship only in males at baseline. However, a note of caution is due here since the occurrences of coronary artery disease during early life is rare and, thus, it is possible that many of the chest pain cases were something else than coronary artery disease (i.e., false positives). Secondly, fatal coronary heart disease would remove persons from the analysis sample.

Less depression was reported by the longitudinal female LTPA profiles increasing to high and moderate LTPA over time at age 60 compared to longitudinal female LTPA profiles that maintained stable low or stable moderate LTPA over time. Our findings only partly agree with the previous evidence of the association between higher PA and lower likelihood of depression [58, 59], as we found that women in the stable moderate LTPA profile had more depression than those in the LTPA profile that increased to moderate over time. One potential reason why we did not find such differences in depression between longitudinal male LTPA profiles than in females may partly be attributed to the lower overall prevalence of depression observed among our male participants (females 18% *versus* males 10%). The inconsistency may also be due to sex-differences in physical activities engaged in. There is evidence that high levels of walking in females and the high levels of strength training in males are most likely associated with the lower prevalence of depression [60]. Because we do not have specific data on the types of PA, we cannot confirm this. However, walking was the most popular type of physical activity among Finnish adults in 2009 and 2017 [61, 62], and we believe this was also most likely the case in 2011 when our participants were last surveyed. Walking as the most popular physical activity engaged in among our participants could explain why we found differences in depression in females but not in males [60].

The key strengths of the present study are a large sample size and the longest, to our knowledge, follow-up time in longitudinal PA profile or trajectory studies published to date. The large sample size with relatively equal sex representation allowed us to conduct separate analyses for females and males. Having the 36-year-long data, we were able to identify the longitudinal LTPA profiles of adult life with a statistical method that has previously only been rarely explored in PA research among working-age adults. With the latent profile analysis, we could uncover unobserved heterogeneity in a population and find substantively meaningful groups of people that are similar in their LTPA behavior. In addition, we used population-based data, which enabled us to capture the entire variation of LTPA behavior in the Finnish adult population. Selection biases are also less likely to be present in the study because the health and behavior survey questionnaires we used consisted of different domains and the response rates of all study waves were high. Given these strengths, we believe that the generalizability of our study findings is very good.

There are also some limitations worth noting. To begin with, it was unfortunate that we were not able to use information on device-measured LTPA, but there were no appropriate PA devices in the mid-1970s when the study was launched. Besides, the LTPA items we used have been shown to be valid and reliable [26, 27]. It is also important to note that our study represents data from the mid-1970s to early 2010s. Results from more recent time points might be different given that the prevalence of LTPA has increased in Finland since the 1970s [63]. Interestingly, this phenomenon can also be seen in our data, with the low LTPA levels being most common at baseline in 1975. In addition, some non-measured confounders, such as major life events, could explain some of the differences we found in longitudinal LTPA profiles within and between sexes [64]. Furthermore, due to the observational nature of the study, we cannot exclude the possibility of reverse causation (i.e., poor lifestyle or health impaired the ability to engage in LTPA). Method-wise, it is also good to note that LTPA profile assignments through the latent profile analysis are probabilistic in nature, not deterministic. Individuals may thus have varying degrees of membership in different latent profiles, but have been assigned to a single profile, which inherently causes some level of uncertainty to which profile they belong. However, the latent profile analysis offers a more nuanced understanding of individual profiles than hierarchical clustering, allowing for an unbiased estimation of the mean and variance of LTPA.

## Conclusions

To conclude, our study has provided new insights into longitudinal LTPA profiles across the working ages. Lower longitudinal LTPA profiles clustered with less advantageous demographics, anthropometrics, lifestyle behaviors, and health in females and males. Our findings further suggest that several different longitudinal LTPA profiles start with low LTPA levels in young adulthood. The divergence of the various profiles begin around age 30 or 40 in both females and males, suggesting these are particularly sensitive windows for changes in LTPA behavior. Within the 36-year follow-up, about half of the participants changed their LTPA levels, for better or worse, which implies the potential to change LTPA behavior during adulthood. Whereas it would be difficult to capture which young adults are at the highest risk of long-term physical inactivity, potential indicators of a low longitudinal LTPA profile during the life course may be a lower education, higher BMI, and regular smoking. Additionally, males are more likely to have low LTPA levels at age 60 than females and, thus, more effort should be put to males’ PA promotion. It seems to be most favorable to belong to the group of people with the highest LTPA levels, though these had the smallest proportions in this study, which means there is a huge opportunity to gain population health benefits from increasing LTPA at the population level. Because of the novel nature of our study, future studies should replicate our results but also plan interventions to support the long-term adherence of sufficient LTPA.

### Supplementary Information

**Additional file 1:** **Supplementary Table 1. **Descriptive statistics of leisure-time physical activity, demographic, anthropometric, lifestyle, and health characteristics by sex at ages 24, 30, 40 and 60.

**Additional file 2:** **Supplementary Table 2. **Means and standard errors of MET hours per day by longitudinal leisure-time physical activity profiles in females and males at four follow-up time points.

**Additional file 3: Supplementary Table 3. **The associations between longitudinal leisure-time physical activity profiles and different demographic, anthropometric and lifestyle characteristics in females.

**Additional file 4: Supplementary Table 4. **The associations between longitudinal leisure-time physical activity profiles and dichotomized health characteristics (yes/no) in females.

**Additional file 5:** **Supplementary Table 5. **The associations between longitudinal leisure-time physical activity profiles and different demographic, anthropometric and lifestyle characteristics in males.

**Additional file 6: Supplementary Table 6. **The associations between longitudinal leisure-time physical activity profiles and dichotomized health characteristics (yes/no) in males.

## Data Availability

Due to the consent given by the participants the older Finnish Twin Cohort and the ease of identifying twins, data cannot be made publicly available. Data are available through the Institute for Molecular Medicine Finland FIMM Data Access Committee (DAC) for authorized researchers who have institutional review board/ethics approval and an institutionally approved study plan. For more details, please contact the FIMM DAC (fimm-dac@helsinki.fi).

## Abbreviations

BIC: Bayesian information criteria
BMI: Body mass index
DAC: Data Access Committee
LTPA: Leisure-time physical activity
LMR: Lo-Mendel-Rubin adjusted test
MET: Metabolic equivalent of task
PA: Physical activity
T2D: Type 2 diabetes
YFS: Young Finns Study
