# Supplementary material for: Longitudinal leisure-time physical activity profiles throughout adulthood and related characteristics: a 36-year follow-up study of the older Finnish Twin Cohort
Source: Int J Behav Nutr Phys Act. 2024 Apr 26;21:47. doi: 10.1186/s12966-024-01600-y (PMC11046842; doi:10.1186/s12966-024-01600-y)
Supplement: Supplementary file 1 — Additional file 1: Supplementary Table 1. Descriptive statistics of leisure-time physical activity, demographic, anthropometric, lifestyle, and health characteristics by sex at ages 24, 30, 40 and 60. [file 12966_2024_1600_MOESM1_ESM.pdf]

Supplementary table 1. Descriptive statistics of leisure-time physical activity, demographic, anthropometric, lifestyle, and health characteristics by sex at ages 24, 30, 40 and 60.

**Variable**	**Females**				**Males**			
	Age 24<br><br>**Mean (SD)**<br>**N**	Age 30<br><br>**Mean (SD)**<br>**N**	Age 40<br><br>**Mean (SD)**<br>**N**	Age 60<br><br>**Mean (SD)**<br>**N**	Age 24<br><br>**Mean (SD)**<br>**N**	Age 30<br><br>**Mean (SD)**<br>**N**	Age 40<br><br>**Mean (SD)**<br>**N**	Age 60<br><br>**Mean (SD)**<br>**N**
Mean LTPA, MET hours/day	2.2 (2.6)<br>2778	2.5 (2.6)<br>2778	3.6 (3.4)<br>2778	3.7 (3.5)<br>2778	3.2 (4.0)<br>1938	3.3 (4.0)<br>1938	3.6 (3.7)<br>1938	3.4 (3.8)<br>1938
Mean age, yrs	23.6 (3.8)<br>2778	29.9 (3.7)<br>2771	38.9 (3.7)<br>2701	60.0 (3.8)<br>2778	24.1 (3.7)<br>1938	30.3 (3.7)<br>1894	39.4 (3.7)<br>1938	60.5 (3.7)<br>1938
Education, categories	3.9 (1.8)<br>2777	4.5 (2.1)<br>2777			3.8 (1.8)<br>1937	4.3 (2.3)<br>1938		
Monthly income, categories	2.9 (1.5)<br>2738				4.1 (1.9)<br>1921			
Mean weight, kg	55.8 (7.7)<br>2761	58.0 (8.5)<br>2767	61.9 (10.8)<br>2763	68.7 (12.7)<br>2741	70.5 (9.3)<br>1932	73.7 (9.9)<br>1931	77.5 (11.0)<br>1937	83.2 (13.2)<br>1901
Mean BMI, kg/m^2	20.9 (2.6)<br>2756	21.7 (3.0)<br>2761	23.2 (3.8)<br>2760	25.8 (4.6)<br>2739	22.5 (2.6)<br>1930	23.5 (2.7)<br>1931	24.7 (3.1)<br>1935	26.7 (3.8)<br>1901
Mean waist circumference, cm				86.7 (12.0)<br>2753				97.6 (10.9)<br>1916
Mean alcohol consumption, grams	127.0 (173.8)<br>2777	121.6 (174.4)<br>2776	164.7 (245.6)<br>2777	186.4 (279.0)<br>2558	322.0 (383.0)<br>1938	321.0 (388.3)<br>1937	388.2 (464.3)<br>1935	488.1 (664.3)<br>1858
Smoking %								
Never	60.9<br>1491	59.4<br>1453	58.8<br>1428	60.3<br>1468	43.4<br>699	41.7<br>672	41.2<br>661	41.8<br>670
Former	11.0<br>270	17.5<br>429	18.6<br>453	24.3<br>592	17.5<br>282	23.3<br>375	27.6<br>442	39.5<br>632
Current	28.1<br>687	23.1<br>564	22.6<br>549	15.3<br>373	39.1<br>629	35.0<br>563	31.2<br>500	18.7<br>300
Mean sleep time, h	7.8 (0.9)<br>2777	7.7 (0.8)<br>2767	7.6 (0.8)<br>2769	7.4 (0.9)<br>2768	7.7 (0.9)<br>1938	7.5 (0.8)<br>1930	7.4 (0.8)<br>1934	7.4 (0.9)<br>1931
Work-related PA, % manual work	41.9<br>2731			49.2<br>2722	40.1<br>1905			51.7<br>1891

Mean sitting time, h				8.5 (2.1)<br>2772				8.8 (2.2)<br>1936
Subjective health status, % poor				28.9<br>2764				33.5<br>1927
High blood pressure, % yes	4.8<br>2752	6.8<br>2766	9.8<br>2715	48.9<br>2768	6.3<br>1919	7.2<br>1934	11.8<br>1902	52.9<br>1927
Coronary artery disease, % yes	1.3<br>2752	0.9<br>2765	1.2<br>2703	2.7<br>2588	1.0<br>1919	0.8<br>1935	1.3<br>1898	7.1<br>1818
T2D, % yes	0.3<br>2752	0.5<br>2765	0.9<br>2699	7.9<br>2770	0.4<br>1919	0.7<br>1935	1.1<br>1896	11.3<br>1931
Depression, % yes				17.9<br>2626				10.4<br>1816

Note. SD=standard deviation; N=number of participants; MET= metabolic equivalent of task; LTPA=leisure-time physical activity; yrs=years; kg=kilogram; BMI=body mass index; m=meter; cm=centimeter; h=hours; PA=physical activity; T2D=type 2 diabetes.
