# Supplementary material for: Longitudinal leisure-time physical activity profiles throughout adulthood and related characteristics: a 36-year follow-up study of the older Finnish Twin Cohort
Source: Int J Behav Nutr Phys Act. 2024 Apr 26;21:47. doi: 10.1186/s12966-024-01600-y (PMC11046842; doi:10.1186/s12966-024-01600-y)
Supplement: Supplementary file 2 — Additional file 2: Supplementary Table 2. Means and standard errors of MET hours per day by longitudinal leisure-time physical activity profiles in females and males at four follow-up time points. [file 12966_2024_1600_MOESM2_ESM.pdf]

Supplementary table 2. Means and standard errors of MET hours per day by longitudinal leisure-time physical activity profiles in females and males at four follow-up time points.

Females					
**Variable**	**Profile 1**<br>*Low increasing moderate*<br>**Mean (SE)**	**Profile 2**<br>*Very low increasing low*<br>**Mean (SE)**	**Profile 3**<br>*High increasing high*<br>**Mean (SE)**	**Profile 4**<br>*Moderate stable*<br>**Mean (SE)**	**Profile 5**<br>*Low stable*<br>**Mean (SE)**
LTPA at age 24	1.4 (0.1)	0.7 (0.0)	6.0 (0.4)	3.4 (0.3)	2.0 (0.2)
LTPA at age 30	1.8 (0.2)	0.8 (0.0)	6.6 (0.4)	3.6 (0.2)	1.9 (0.1)
LTPA at age 40	4.5 (0.2)	1.6 (0.2)	8.6 (0.5)	3.9 (0.2)	1.7 (0.2)
LTPA at age 60	4.7 (0.2)	2.1 (0.1)	8.9 (0.5)	3.8 (0.3)	1.5 (0.1)
**Males**					
**Variable**	**Profile 1**<br>*Low stable very low*<br>**Mean (SE)**	**Profile 2**<br>*Very low stable*<br>**Mean (SE)**	**Profile 3**<br>*Low increasing moderate*<br>**Mean (SE)**	**Profile 4**<br>*High fluctuating high*<br>**Mean (SE)**	**Profile 5**<br>*Moderate decreasing low*<br>**Mean (SE)**
LTPA at age 24	1.6 (0.1)	0.4 (0.0)	1.5 (0.1)	8.1 (0.6)	4.5 (0.4)
LTPA at age 30	1.3 (0.2)	0.6 (0.1)	2.0 (0.2)	9.0 (0.6)	3.9 (0.3)
LTPA at age 40	1.3 (0.2)	1.2 (0.2)	4.3 (0.3)	7.5 (0.4)	3.2 (0.2)
LTPA at age 60	1.2 (0.1)	1.0 (0.1)	4.3 (0.4)	7.7 (0.4)	2.4 (0.2)

Note. MET=metabolic equivalent of task; LTPA=leisure-time physical activity quantified as MET hours per day; SE=standard error
