# Supplementary material for: Longitudinal leisure-time physical activity profiles throughout adulthood and related characteristics: a 36-year follow-up study of the older Finnish Twin Cohort
Source: Int J Behav Nutr Phys Act. 2024 Apr 26;21:47. doi: 10.1186/s12966-024-01600-y (PMC11046842; doi:10.1186/s12966-024-01600-y)
Supplement: Supplementary file 3 — Additional file 3: Supplementary Table 3. The associations between longitudinal leisure-time physical activity profiles and different demographic, anthropometric and lifestyle characteristics in females. [file 12966_2024_1600_MOESM3_ESM.pdf]

Supplementary table 3. The associations between longitudinal leisure-time physical activity profiles and different demographic, anthropometric and lifestyle characteristics in females. Age, weight, BMI, waist circumference and alcohol consumption are analyzed as continuous variables and their units are given in the table, other variables are treated as categorized variables. Chi square and overall p-values are given for the comparison between all profiles. Groupwise comparisons indicate individual tests between distinct profiles and their significances.

**Variable**	**Profile 1** *Low increasing moderate* **Mean (SE)**	**Profile 2** *Very low increasing low* **Mean (SE)**	**Profile 3** *High increasing high* **Mean (SE)**	**Profile 4** *Moderate stable* **Mean (SE)**	**Profile 5** *Low stable* **Mean (SE)**	$\chi^2(4)$	**Overall *p*-value**	**Groupwise comparison**
Mean age[1], yrs	23.5 (0.2)	24.0 (0.2)	23.3 (0.3)	23.4 (0.2)	23.8 (0.2)	7.89	0.096	
Mean age[2], yrs	29.7 (0.2)	30.3 (0.2)	29.6 (0.3)	29.6 (0.2)	30.0 (0.2)	7.94	0.094	
Mean age[3], yrs	38.8 (0.2)	39.4 (0.2)	38.7 (0.3)	38.7 (0.2)	39.1 (0.2)	7,85	0.097	
Mean age[4], yrs	59.9 (0.2)	60.4 (0.2)	59.7 (0.3)	59.7 (0.2)	60.2 (0.2)	48.86	0.065	
Education[1], categories	3.9 (0.1)	3.6 (0.1)	3.8 (0.1)	4.1 (0.1)	3.9 (0.1)	14.53	0.006	2 < 1,4,5
Education[2], categories	4.3 (0.1)	3.9 (0.1)	4.3 (0.1)	4.4 (0.1)	4.3 (0.1)	17.56	0.002	2 < 1,3,4,5
Monthly income[1], categories	2.9 (0.1)	2.7 (0.1)	3.0 (0.1)	3.0 (0.1)	3.1 (0.1)	8.69	0.069	
Mean weight[1], kg	54.9 (0.3)	57.0 (0.4)	54.5 (0.5)	55.9 (0.4)	56.5 (0.5)	23.28	< 0.001	1 < 2,5; 3 < 2,4,5
Mean weight[2], kg	56.9 (0.4)	59.6 (0.5)	55.8 (0.5)	57.8 (0.4)	59.0 (0.5)	40.99	< 0.001	1 < 2,5; 2 > 4; 3 < 2,4,5
Mean weight[3], kg	59.9 (0.4)	64.2 (0.6)	58.3 (0.6)	62.2 (0.6)	63.7 (0.6)	70.05	< 0.001	1 < 2,4,5; 2 > 4; 3 < 1,2,4,5
Mean weight[4], kg	66.5 (0.5)	71.1 (0.7)	63.8 (0.8)	68.7 (0.6)	71.9 (0.8)	82.29	< 0.001	1 < 2,4,5; 3 < 1,2,4,5; 4 < 2,5
Mean BMI[1], kg/m^2	20.5 (0.1)	21.3 (0.1)	20.4 (0.1)	20.8 (0.1)	21.2 (0.2)	30.48	< 0.001	1 < 2,5; 4 < 2,5; 3 < 2,5

Mean BMI[2], kg/m^2	21.3 (0.2)	22.3 (0.3)	20.9 (0.3)	21.5 (0.2)	22.2 (0.3)	53.92	< 0.001	1 < 2,5; 3 < 2,4,5; 4 < 2,5
Mean BMI[3], kg/m^2	22.4 (0.1)	24.0 (0.2)	21.9 (0.2)	23.2 (0.2)	23.9 (0.2)	83.96	< 0.001	1 < 2,4,5; 3 < 1,2,4,5; 4 < 2,5
Mean BMI[4], kg/m^2	25.0 (0.2)	26.8 (0.3)	24.1 (0.3)	25.6 (0.2)	27.0 (0.3)	85.89	< 0.001	1 < 2,5; 3 < 1,2,4,5; 4 < 2,5
Mean waist circumference[4], cm	84.1 (0.5)	89.3 (0.6)	82.3 (0.9)	86.2 (0.6)	90.4 (0.7)	95.31	< 0.001	1 < 2,4,5; 3 < 2,4,5; 4 <2,5
Mean alcohol consumption[1], grams	135.1 (7.6)	123.1 (7.7)	121.9 (11.3)	131.1 (11.0)	116.9 (10.3)	2.59	0.628	
Mean alcohol consumption[2], grams	124.1 (8.1)	126.1 (8.2)	117.9 (11.0)	114.8 (9.1)	123.1 (11.0)	1.17	0.883	
Mean alcohol consumption[3], grams	173.7 (11.9)	179.8 (13.6)	155.0 (16.0)	160.0 (12.4)	146.3 (12.4)	3.94	0.414	
Mean alcohol consumption[4], grams	191.1 (12.3)	215.0 (17.6)	189.5 (20.0)	165.9 (14.7)	172.7 (15.4)	4.99	0.288	
Smoking[1] %						21.11	0.007	
Never	59.9 (2.5)	55.7 (2.7)	62.7 (3.9)	68.2 (2.8)	58.6 (3.1)			1,2 < 4; 5 < 4
Former	8.6 (1.4)	11.8 (1.7)	15.6 (2.9)	10.7 (1.8)	11.9 (2.0)			1 < 3
Current	31.5 (2.3)	32.5 (2.5)	21.7 (3.2)	21.1 (2.4)	29.6 (2.9)			3,4 < 1,2; 4 < 5
Smoking[2] %						12.10	0.147	
Never	58.2 (2.5)	53.9 (2.7)	63.9 (3.8)	65.3 (2.8)	57.9 (3.1)			
Former	17.7 (1.9)	18.3(2.1)	16.2 (2.8)	15.7 (2.1)	19.3 (2.4)			
Current	24.0 (2.2)	27.8 (2.4)	19.9 (3.0)	19.0 (2.3)	22.8 (2.6)			
Smoking[3] %						17.18	0.028	
Never	57.2 (2.5)	52.9 (2.8)	63.9 (3.9)	65.2 (2.8)	57.3 (3.1)			2 < 3,4
Former	20.1 (2.0)	18.5 (2.1)	14.7 (2.8)	18.0 (2.3)	19.3 (2.5)			
Current	22.7 (2.1)	28.6 (2.4)	21.4 (3.2)	16.9 (2.2)	23.4 (2.7)			2 > 4
Smoking[4] %						15.65	0.048	
Never	59.1 (2.5)	56.2 (2.7)	66.4 (3.8)	65.8 (2.8)	57.3 (3.1)			

Former	26.2 (2.2)	23.3 (2.3)	20.6 (3.1)	22.0 (2.4)	27.2 (2.7)			
Current	14.7 (1.8)	20.5 (2.1)	13.0 (2.6)	12.2 (2.0)	15.4 (2.2)			1,3,4 < 2
Mean sleep time[1], h	7.9 (0.0)	7.9 (0.0)	7.8 (0.1)	7.8 (0.0)	7.8 (0.0)	3.18	0.528	
Mean sleep time[2], h	7.5 (0.1)	7.7 (0.1)	7.4 (0.1)	7.5 (0.1)	7.4 (0.1)	11.42	0.022	1,3,4,5 < 2
Mean sleep time[3], h	7.1 (0.1)	7.2 (0.1)	7.0 (0.1)	7.3 (0.1)	7.1 (0.1)	4.42	0.352	
Mean sleep time[4], h	6.8 (0.1)	6.7 (0.1)	6.5 (0.1)	6.7 (0.1)	6.9 (0.1)	3.59	0.464	
Work-related PA[1], % manual work	40.3 (2.4)	42.5 (2.6)	48.0 (3.7)	42.5 (2.7)	39.8 (2.9)	3.93	0.416	
Work-related PA[4], % manual work	48.8 (2.4)	52.3 (2.7)	52.9 (3.7)	49.0 (2.7)	45.2 (2.9)	4.01	0.405	
Mean sitting time[4], h	8.4 (0.1)	8.5 (0.1)	8.2 (0.1)	8.4 (0.1)	8.7 (0.1)	12.55	0.016	1,3 < 5; 2 > 3

Note. Measurement time points [1]=age 24; [2]=age 30; [3]=age 40 and [4]=age 60. The *p*-value < 0.001 corresponds to a multiple-test (45 tests) corrected Bonferroni *p*-value < 0.05.

χ^2=Chi-square; yrs=years; kg=kilogram; BMI=body mass index; m=meter; cm=centimeter; h=hours; PA=physical activity
