# Supplementary material for: Longitudinal leisure-time physical activity profiles throughout adulthood and related characteristics: a 36-year follow-up study of the older Finnish Twin Cohort
Source: Int J Behav Nutr Phys Act. 2024 Apr 26;21:47. doi: 10.1186/s12966-024-01600-y (PMC11046842; doi:10.1186/s12966-024-01600-y)
Supplement: Supplementary file 4 — Additional file 4: Supplementary Table 4. The associations between longitudinal leisure-time physical activity profiles and dichotomized health characteristics (yes/no) in females. [file 12966_2024_1600_MOESM4_ESM.pdf]

Supplementary table 4. The associations between longitudinal leisure-time physical activity profiles and dichotomized health characteristics (yes/no) in females.

**Variable**	**Profile 1**<br>*Low increasing moderate*<br>**Mean (SE)**	**Profile 2**<br>*Very low increasing low*<br>**Mean (SE)**	**Profile 3**<br>*High increasing high*<br>**Mean (SE)**	**Profile 4**<br>*Moderate stable*<br>**Mean (SE)**	**Profile 5**<br>*Low stable*<br>**Mean (SE)**	$\chi^2(4)$	**Overall *p*-value**	**Groupwise comparison**
Subjective health status[4], % poor	19.3 (1.9)	35.0 (2.4)	14.3 (2.5)	30.5 (2.5)	41.4 (2.8)	68.25	< 0.001	1 < 2,4,5; 3 < 2,4,5; 4 < 5
High blood pressure[1], % yes	4.2 (0.9)	6.1 (1.2)	3.0 (1.2)	3.8 (1.1)	6.3 (1.3)	6.28	0.179	
High blood pressure[2], % yes	4.8 (1.0)	7.6 (1.3)	4.6 (1.5)	6.7 (1.3)	9.7 (1.6)	9.19	0.057	
High blood pressure[3], % yes	6.6 (1.2)	11.8 (1.7)	6.5 (1.8)	9.5 (1.6)	14.2 (1.9)	116.92	0.002	1,3 < 5; 1 < 2
High blood pressure[4], % yes	44.7 (2.3)	52.8 (2.6)	40.1 (3.6)	47.5 (2.7)	56.9 (2.9)	20.17	< 0.001	1,3 < 2,5; 4 < 5
Coronary artery disease[1], % yes	2.2 (0.6)	0.9 (0.6)	1.8 (0.9)	0.8 (0.5)	0.7 (0.6)	3.63	0.459	
Coronary artery disease[2], % yes	1.6 (0.5)	1.0 (0.5)	0.9 (0.7)	0.4 (0.4)	0.4 (0.4)	2.93	0.570	
Coronary artery disease[3], % yes	1.5 (0.6)	1.1 (0.6)	0.9 (0.7)	0.8 (0.5)	1.5 (0.8)	0.98	0.913	
Coronary artery disease[4], % yes	1.9 (0.7)	3.3 (1.0)	1.3 (0.9)	2.1 (0.8)	4.7 (1.2)	7.92	0.095	
T2D[1], % yes	0.1 (0.2)	0.0 (0.0)	0.5 (0.5)	0.1 (0.2)	1.1 (0.5)	91.95	< 0.001	2 < 1,3,4,5
T2D[2], % yes	0.3 (0.3)	0.0 (0.0)	0.0 (0.0)	0.5 (0.4)	1.7 (0.7)	193.16	< 0.001	2,3 < 1,4,5
T2D[3], % yes	0.6 (0.4)	1.2 (0.6)	0.0 (0.0)	0.8 (0.5)	1.4 (0.6)	104.75	< 0.001	3 < 1,2,4,5
T2D[4], % yes	6.0 (1.2)	10.1 (1.6)	4.1 (1.4)	7.4 (1.4)	10.9 (1.7)	13.18	0.010	1 ,3 < 2,5
Depression[4], % yes	12.8 (1.7)	20.1 (2.1)	14.7 (2.7)	19.9 (2.2)	22.5 (2.5)	13.22	0.010	1 < 2,4,5; 3 < 5

Note. Measurement time points [1]=age 24; [2]=age 30; [3]=age 40 and [4]=age 60. The p-value < 0.001 corresponds to a multiple-test (45 tests) corrected Bonferroni p-value < 0.05.

χ^2=Chi-square; T2D=type 2 diabetes
