# Supplementary material for: Longitudinal leisure-time physical activity profiles throughout adulthood and related characteristics: a 36-year follow-up study of the older Finnish Twin Cohort
Source: Int J Behav Nutr Phys Act. 2024 Apr 26;21:47. doi: 10.1186/s12966-024-01600-y (PMC11046842; doi:10.1186/s12966-024-01600-y)
Supplement: Supplementary file 5 — Additional file 5: Supplementary Table 5. The associations between longitudinal leisure-time physical activity profiles and different demographic, anthropometric and lifestyle characteristics in males. [file 12966_2024_1600_MOESM5_ESM.pdf]

Supplementary table 5. The associations between longitudinal leisure-time physical activity profiles and different demographic, anthropometric and lifestyle characteristics in males. Age, weight, BMI, waist circumference and alcohol consumption are analyzed as continuous variables and their units are given in the table, other variables are treated as categorized variables. Chi square and overall p-values are given for the comparison between all profiles. Groupwise comparisons indicate individual tests between distinct profiles and their significances.

**Variable**	**Profile 1** *Low stable very low* **Mean (SE)**	**Profile 2** *Very low stable* **Mean (SE)**	**Profile 3** *Low increasing moderate* **Mean (SE)**	**Profile 4** *High fluctuating high* **Mean (SE)**	**Profile 5** *Moderate decreasing low* **Mean (SE)**	$\chi^2(4)$	**Overall *p*-value**	**Groupwise comparison**
Mean age[1], yrs	24.3 (0.2)	24.7 (0.3)	24.2 (0.2)	23.6 (0.2)	24.0 (0.2)	8.13	0.087	
Mean age[2], yrs	30.5 (0.2)	31.0 (0.3)	30.4 (0.2)	29.8 (0.2)	30.2 (0.2)	8.94	0.063	
Mean age[3], yrs	39.6 (0.2)	39.9 (0.3)	39.5 (0.2)	38.9 (0.2)	39.2 (0.2)	8.55	0.073	
Mean age[4], yrs	60.7 (0.2)	61.1 (0.3)	60.6 (0.2)	60.0 (0.2)	60.4 (0.2)	9.00	0.061	
Education[1], categories	3.68 (0.1)	3.11 (0.1)	3.78 (0.1)	4.19 (0.1)	3.96 (0.1)	47.67	< 0.001	1 < 4; 2 < 1,3,4,5; 3 < 4
Education[2], categories	4.09 (0.1)	3.31 (0.2)	4.27 (0.1)	4.85 (0.2)	4.56 (0.2)	62.07	< 0.001	1 < 4,5; 2 < 1,3,4,5; 3 < 4
Monthly income[1], categories	4.19 (0.1)	4.37 (0.1)	4.17 (0.1)	3.90 (0.1)	4.17 (0.1)	5.68	0.224	
Mean weight[1], kg	71.4 (0.5)	72.4 (0.9)	69.7 (0.5)	69.4 (0.5)	70.7 (0.6)	14.43	0.006	1,2 > 3
Mean weight[2], kg	75.0 (0.5)	75.7 (0.9)	72.5 (0.5)	72.3 (0.5)	73.9 (0.6)	21.00	< 0.001	1,2 > 3,4
Mean weight[3], kg	79.5 (0.6)	79.7 (1.0)	76.1 (0.5)	75.6 (0.6)	77.9 (0.7)	29.42	< 0.001	1,2 > 3,4; 4 < 5
Mean weight[4], kg	86.2 (0.7)	85.5 (1.2)	81.0 (0.6)	80.7 (0.8)	84.0 (0.9)	38.53	< 0.001	1,2,5 > 3,4
Mean BMI[1], kg/m^2	22.8 (0.1)	23.1 (0.2)	22.3 (0.1)	22.2 (0.1)	22.6 (0.2)	17.75	0.001	1,2 > 3,4

Mean BMI[2], kg/m^2	23.9 (0.1)	24.1 (0.3)	23.2 (0.1)	23.1 (0.1)	23.6 (0.2)	24.65	< 0.001	1,2 > 3,4
Mean BMI[3], kg/m^2	25.3 (0.2)	25.4 (0.3)	24.3 (0.2)	24.2 (0.2)	24.9 (0.2)	33.91	< 0.001	1,2 > 3,4; 4 < 5
Mean BMI[4], kg/m^2	27.6 (0.2)	27.4 (0.4)	26.0 (0.2)	25.8 (0.2)	26.9 (0.3)	44.68	< 0.001	1,2,5 > 3,4
Mean waist circumference[4], cm	100.8 (0.6)	99.9 (1.0)	95.9 (0.5)	93.4 (0.6)	98.4 (0.7)	87.99.0	< 0.001	1 > 3,4,5; 2 > 3,4; 3 > 4; 3,4 < 5
Mean alcohol consumption[1], grams	326.7 (19.6)	359.5 (34.6)	332.1 (21.0)	298.8 (23.5)	309.1 (21.7)	2.68	0.612	
Mean alcohol consumption[2], grams	337.3 (20.2)	389.0 (36.9)	315.1 (21.7)	278.8 (21.1)	317.8 (22.2)	7.35	0.119	
Mean alcohol consumption[3], grams	416.5 (23.5)	465.8 (59.7)	332.4 (20.1)	394.7 (28.9)	397.4 (29.3)	10.03	0.040	3 < 1,2
Mean alcohol consumption[4], grams	535.7 (38.4)	475.0 (46.8)	454.2 (31.9)	524.9 (49.0)	454.0 (38.9)	3.71	0.447	
Smoking[1] %						29.48	< 0.001	
Never	40.3 (2.8)	36.2 (4.8)	41.7 (2.9)	61.9 (3.5)	37.7 (3.4)			1,2,3,5 < 4
Former	17.2 (2.1)	17.9 (3.7)	19.1 (2.3)	13.2 (2.4)	19.0 (2.7)			
Current	42.5 (2.8)	45.8 (4.9)	39.3 (2.9)	24.9 (3.1)	43.3 (3.4)			4 < 1,2,3,5
Smoking[2] %						40.13	< 0.001	
Never	36.2 (2.8)	33.9 (4.7)	40.8 (2.9)	60.3 (3.5)	38.0 (3.4)			1,2,3,5 < 4
Former	21.0 (2.3)	20.2 (3.9)	24.5 (2.5)	20.2 (2.7)	27.9 (3.1)			
Current	42.7 (2.9)	45.9 (4.9)	34.7 (2.8)	19.6 (2.8)	34.1 (3.2)			4 < 1,2,3,5; 2 > 3,5
Smoking[3] %						41.89	< 0.001	
Never	36.7 (2.8)	33.3 (4.7)	40.0 (2.9)	58.7 (3.5)	37.6 (3.4)			1,2,3,5 < 4
Former	23.9 (2.4)	23.6 (4.1)	30.8 (2.8)	22.9 (2.9)	32.7 (3.2)			1,4 < 5
Current	39.4 (2.8)	43.1 (4.7)	29.2 (2.7)	18.4 (2.7)	29.7 (3.1)			3,4,5 < 1,2; 4 < 3,5
Smoking[4] %						31.26	< 0.001	

Never	37.2 (2.8)	32.9 (4.6)	40.3 (2.9)	58.6 (3.5)	39.5 (3.4)			1,2,3,5 < 4
Former	38.7 (2.8)	46.0 (4.8)	43.9 (2.9)	28.9 (3.2)	40.4 (3.4)			4 < 1,2,3,5
Current	24.1 (2.4)	21.0 (4.0)	15.8 (2.3)	12.5 (2.3)	20.0 (2.7)			3,4 < 1
Mean sleep time[1], h	7.6 (0.0)	7.6 (0.1)	7.6 (0.0)	7.6 (0.1)	7.7 (0.1)	1.90	0.755	
Mean sleep time[2], h	7.1 (0.1)	7.0 (0.1)	7.0 (0.1)	7.1 (0.1)	7.1 (0.1)	1.70	0.790	
Mean sleep time[3], h	6.8 (0.1)	6.7 (0.1)	6.6 (0.1)	6.8 (0.1)	6.8 (0.1)	3.66	0.454	
Mean sleep time[4], h	6.7 (0.1)	6.7 (0.1)	6.7 (0.1)	6.7 (0.1)	6.8 (0.1)	1.09	0.896	
Work-related PA[1], % manual work	58.6 (0.0)	65.3 (0.0)	58.7 (0.0)	61.3 (0.0)	60.4 (0.0)	3.70	0.448	
Work-related PA[4], % manual work	53.4 (2.6)	61.1 (4.4)	50.2 (2.6)	54.3 (3.3)	46.3 (3.2)	8.45	0.076	
Mean sitting time[4], h	8.9 (0.1)	9.0 (0.2)	8.8 (0.1)	8.3 (0.1)	8.8 (0.1)	15.71	0.003	4 < 1,2,3,5

Note. Measurement time points [1]=age 24; [2]=age 30; [3]=age 40 and [4]=age 60. The *p*-value < 0.001 corresponds to a multiple-test (45 tests) corrected Bonferroni *p*-value < 0.05.

χ^2=Chi-square; yrs=years; kg=kilogram; BMI=body mass index; m=meter; cm=centimeter; h=hours; PA=physical activity
