# Supplementary material for: Longitudinal leisure-time physical activity profiles throughout adulthood and related characteristics: a 36-year follow-up study of the older Finnish Twin Cohort
Source: Int J Behav Nutr Phys Act. 2024 Apr 26;21:47. doi: 10.1186/s12966-024-01600-y (PMC11046842; doi:10.1186/s12966-024-01600-y)
Supplement: Supplementary file 6 — Additional file 6: Supplementary Table 6. The associations between longitudinal leisure-time physical activity profiles and dichotomized health characteristics (yes/no) in males. [file 12966_2024_1600_MOESM6_ESM.pdf]

Supplementary table 6. The associations between longitudinal leisure-time physical activity profiles and dichotomized health characteristics (yes/no) in males.

Variable	Profile 1 *Low stable very low* Mean (SE)	Profile 2 *Very low stable* Mean (SE)	Profile 3 *Low increasing moderate* Mean (SE)	Profile 4 *High fluctuating high* Mean (SE)	Profile 5 *Moderate decreasing low* Mean (SE)	$\chi^2(4)$	Overall *p*-value	Groupwise comparison
Subjective health status[4], % poor	44.0 (2.6)	37.0 (4.2)	27.1 (2.4)	19.6 (2.6)	39.3 (3.0)	46.74	< 0.001	1,2 > 3,4; 3 > 4; 3,4 < 5
High blood pressure[1], % yes	4.8 (1.2)	8.0 (2.3)	5.5 (1.2)	6.2 (1.5)	8.5 (1.8)	4.04	0.400	
High blood pressure[2], % yes	8.6 (1.4)	9.2 (2.5)	6.7 (1.3)	7.3 (1.7)	5.6 (1.5)	2.93	0.570	
High blood pressure[3], % yes	11.6 (1.7)	12.0 (3.0)	9.9 (1.6)	12.3 (2.1)	14.0 (2.2)	2.00	0.736	
High blood pressure[4], % yes	59.0 (2.6)	48.3 (4.5)	50.2 (2.6)	43.1 (3.1)	58.7 (3.2)	19.17	< 0.001	1 > 2,3,4; 4 < 5
Coronary artery disease[1], % yes	2.3 (0.7)	0.5 (0.7)	1.1 (0.5)	0.0 (0.0)	0.5 (0.5)	114.86	< 0.001	1,2,3,5 > 4
Coronary artery disease[2], % yes	1.2 (0.5)	0.6 (0.7)	0.7 (0.4)	0.6 (0.5)	0.8 (0.6)	0.76	0.943	
Coronary artery disease[3], % yes	2.2 (0.7)	1.3 (1.0)	1.1 (0.5)	1.0 (0.6)	0.7 (0.6)	3.29	0.511	
Coronary artery disease[4], % yes	8.0 (1.5)	11.8 (2.9)	6.2 (1.3)	3.6 (1.2)	8.4 (1.7)	9.04	0.060	
T2D[1], % yes	0.1 (0.2)	0.0 (0.0)	1.0 (0.5)	0.0 (0.0)	0.5 (0.5)	165.08	< 0.001	2,4 < 1,3,5
T2D[2], % yes	0.0 (0.2)	1.4 (1.0)	0.6 (0.4)	0.2 (0.4)	1.8 (0.8)	2.93	0.569	
T2D[3], % yes	0.7 (0.4)	2.1 (1.2)	0.8 (0.5)	0.6 (0.5)	1.8 (0.8)	3.13	0.536	
T2D[4], % yes	14.0 (1.8)	19.0 (3.5)	8.0 (1.5)	6.1 (1.5)	14.1 (2.2)	20.35	< 0.001	3,4 < 1,2,5
Depression[4], % yes	9.3 (1.6)	10.1 (2.8)	10.8 (1.6)	8.8 (1.8)	12.3 (2.2)	1.84	0.765	

Note. Measurement time points [1]=age 24; [2]=age 30; [3]=age 40 and [4]=age 60. The *p*-value < 0.001 corresponds to a multiple-test (45 tests) corrected Bonferroni *p*-value < 0.05.

χ^2=Chi-square; T2D=type 2 diabetes
